# Immunohistochemical Profiling of Immune Checkpoints in Chronic Hepatitis B Liver Tissue

**DOI:** 10.3390/pathogens14060596

**Published:** 2025-06-18

**Authors:** João Panão-Costa, Rui Caetano Oliveira, Paulo Teixeira, Francisco Caramelo, Maria Augusta Cipriano, Olga Borges, Armando Carvalho

**Affiliations:** 1 CNC-UC—Center for Neuroscience and Cell Biology, University of Coimbra, 3004-504 Coimbra, Portugal; jpanao94@gmail.com; 2 CIBB—Center for Innovative Biomedicine and Biotechnology, University of Coimbra, 3004-504 Coimbra, Portugal; fcaramelo@fmed.uc.pt; 3 Faculty of Pharmacy, University of Coimbra, 3000-548 Coimbra, Portugal; 4 Department of Pathology, Centro Hospitalar e Universitário de Coimbra, 3000-075 Coimbra, Portugal; ruipedrocoliveira@hotmail.com (R.C.O.); ptbongo@gmail.com (P.T.); augustacipriano@gmail.com (M.A.C.); 5 Institute of Histology, Faculty of Medicine, University of Coimbra, 3000-548 Coimbra, Portugal; 6 Institute for Clinical and Biomedical Research (iCBR): Environment, Genetics and Oncobiology (CIMAGO), Faculty of Medicine, University of Coimbra, 3000-548 Coimbra, Portugal; 7 Coimbra Health School, Polytechnic University of Coimbra, 3045-093 Coimbra, Portugal; 8 Centro Hospitalar e Universitário de Coimbra, 3004-561 Coimbra, Portugal; aspcarvalho@gmail.com; 9 Faculty of Medicine, University of Coimbra, 3004-504 Coimbra, Portugal

**Keywords:** chronic hepatitis B, immune checkpoint receptors, immune checkpoint inhibitors, immune checkpoint blockade, immune exhaustion, immunotherapy, immunohistochemistry, TIM-3, PD-1, CTLA-4

## Abstract

Chronic hepatitis B (CHB) remains a significant global health concern due to complications like cirrhosis and liver cancer. Immune cell exhaustion, characterized by increased suppressive molecules and inhibitory receptors, represents a critical feature of CHB. Understanding the mechanisms of hepatic immune exhaustion in CHB patients is imperative for the development of effective therapeutic interventions. In this study, we investigated the expression levels and histological distribution of various immune checkpoint receptors and ligands in liver biopsies obtained from CHB patients. Additionally, we aimed to evaluate potential concurrent overexpression of specific receptors and their association with clinical parameters such as ALT levels. Our analysis revealed that PD-1, PD-L1, CTLA-4, TIM-3, GAL-9, CD272, TIGIT, and 2B4 exhibited predominant localization in portal tracts and sinusoids. Furthermore, we observed a correlation between the expression of PD-1, TIM-3, and GAL-9 with ALT levels in CHB patients. Additionally, a strong relationship was identified between the expression of CD272 and TIGIT, as well as between GAL-9 and CTLA-4 within the studied population. Our findings underscore the significance of the TIM-3:GAL-9 pathway in the immunopathogenesis of CHB. This detailed analysis sets the stage for future combined immunotherapy strategies aimed at leveraging checkpoint receptors to enhance clinical outcomes.

## 1. Introduction

Chronic hepatitis B (CHB) is a hepatic disease caused by hepatitis B virus (HBV) infection, constituting a significant global health concern affecting a large population worldwide. Persistent HBV infection can lead to chronic inflammation, hepatic impairment, and progress to severe complications like cirrhosis and hepatocellular carcinoma (HCC) [1]. Current therapeutic strategies aim at sustaining viral replication suppression, halting disease progression, and reducing liver-associated risks. These approaches involve nucleos(t)ide analogs (NAs), which hinder HBV reverse transcriptase and viral replication, alongside pegylated interferon-alpha (PEG-IFNα), an immunomodulator amplifying host immune responses against the infection [2]. Despite treatment efficacy, complete HBV eradication remains problematic, as the virus employs evasion tactics, escaping immune clearance and establishing a resilient liver reservoir for persistent replication (intranuclear cccDNA) [3]. Strategically merging immunomodulation with direct-acting antivirals, capable of concurrently impeding viral replication and reducing antigen load, could prove essential in attaining a functional cure [4]. Throughout CHB disease course, patients’ immune system suffers progressive T cell dysfunction and exhaustion, impairing proliferation, cytokine production, and cytotoxicity against infected cells [5]. In immune-exhausted individuals, elevated levels of suppressive cells and molecules are prevalent, including regulatory T cells, IL-10, TGF-β, and inhibitory receptors, like PD-1 and CTLA-4 [6,7]. Recent advances in immunotherapy have shown potential in leveraging the immune system to eliminate infected cells. Notably, immune checkpoint inhibitors counteract inhibitory signals hindering effective antiviral immune responses [8]. To develop an immune checkpoint inhibitor strategy for CHB, understanding the disease’s pathogenesis, specifically immune cell exhaustion mechanisms, is pivotal.

Despite the systemic consequences of HBV-driven immune exhaustion, evaluating inhibitory receptor expression in HBV-specific T cells is optimally conducted in intrahepatic tissue, where cell abundance aligns with the robust tolerogenic milieu. The limited availability of studies with conclusive results can be attributed mainly to the difficulty researchers face in obtaining access to liver biopsies from HBV patients. Moreover, the assessment of immune checkpoints in each liver sample typically encompasses only a few immune checkpoints. This practice curtails the ability to draw comprehensive conclusions regarding potential synergistic interactions among distinct immune checkpoints.

Our objective is to assess the expression levels of immune checkpoint receptors in liver biopsies from patients with CHB, as well as their histological distribution, and to ascertain whether certain receptors are concurrently overexpressed. Additionally, we aim to investigate whether this overexpression correlates with the clinical parameters of the patients. To accomplish this, we will perform a retrospective analysis of liver biopsies obtained from individuals with CHB. This analysis will involve the use of immunohistochemistry (IHC) to examine the presence of immune checkpoints in liver tissue cells extracted from the biopsy samples. The immunohistochemical analysis will encompass a range of inhibitory cellular receptors, including well-studied ones like PD-1, CTLA-4 and TIM-3, as well as LAG-3, TIGIT, KLRG-1, 2B4, CD272, CD160, PD-L1, PD-L2, and GAL-9, for which limited information is available in the existing literature.

## 2. Materials and Methods

### 2.1. The Specimens

Specimens of liver tissues were obtained by percutaneous or surgical biopsy from 30 hepatitis B patients (including 10 with HCC) at Coimbra University Hospital Centre, Coimbra, Portugal (Table 1). All studies were conducted in accordance with institutional ethical guidelines and were approved by the Ethics Committee for Health of the Coimbra University Hospital Centre (approval number 358.OBS.SF.066-2022). Patients were identified resorting to the clinical database, and the analyses were only performed in samples with enough tissue, ensuring that the study would not completely exhaust the sample. CHB was defined as persistent hepatitis B surface antigen (HBsAg) positivity for at least six months, with detectable HBV-DNA in serum, in accordance with EASL 2017 guidelines [3].

The histological activity index (HAI) was evaluated following the criteria outlined by Ishak and colleagues [9], incorporating both grading and staging scores. Tumor staging was determined according to the guidelines established at the Chengdu conference [10], while tumor grading was based on the 2019 WHO classification of tumors of the digestive system. The Chengdu conference staging system considered factors such as tumor size, lobar distribution, vascular thrombosis, lymph node metastasis, distant metastasis, and Child–Pugh staging. None of the patients received radiation or chemotherapy prior to surgery.

### 2.2. Immunohistochemical Staining

The samples were available in the form of formalin-fixed paraffin-embedded tissue. Immunohistochemical (IHC) study was performed into 4 µm tissue sections on Superfrost Plus Slides (Thermo Fisher Scientific^®^ Plus, Braunschweig, Germany). To promote slide-tissue adhesion, all glass slides were preheated at 60 °C in an oven prior to IHC staining for 30 min. Immunostaining was carried out on Ventana Benchmark Ultra platform (Ventana Medical System, Tucson, AZ, USA). Antigen retrieval from previous antibodies was carried out with Cell Condition 1 Tris-based buffer (CC1, Ventana Medical System, Tucson, AZ, USA) for 32 min at 95 °C. Primary antibodies anti-PD-1 (NAT105), anti-PD-L1 (EPR19759), anti-PD-L2 (EPR25200-50), anti-CTLA-4 (CAL49), anti-TIM-3 (EPR22241), anti-LAG-3 (EPR20261), anti-TIGIT (BLR047F), anti-KLRG-1 (Polyclonal), anti-2B4 (EPR23692-33), anti-GAL-9 (EPR22214), anti-CD272 (EPR22224-271), and anti-CD160 (Polyclonal) were purchased from Abcam (Cambridge, UK) and incubated during 40 min at 36 °C (except for anti-2B4: 48 min at 36 °C) at the final dilutions of 1:100, 1:60, 1:500, 1:300, 1:750, 1:750, 1:750, 1:800, 1:50, 1:1000, 1:750, 1:50, respectively. Endogenous peroxidase activity was blocked with 3% hydrogen peroxide from OptiView DAB IHC Detection Kit (Ventana Medical System, Tucson, AZ, USA) for 4 min at room temperature, as per manufacturer recommendations. Primary antibodies were then detected with OptiView DAB IHC Detection Kit (Ventana Medical System, Tucson, AZ, USA) as an indirect, biotin-free system to detect primary antibodies, as per manufacturer recommendations. Antibody–antigen binding was visualized with 3,3′-diaminobenzidine tetrahydrochloride (DAB) chromogen from OptiView DAB IHC Detection Kit (Ventana Medical System, Tucson, AZ, USA), as a brown precipitate, as per manufacturer recommendations. Tissue sections were then nuclear counterstained with hematoxylin and mounted with synthetic mounting media Entellan (Merck, Darmstadt, Germany). The expression levels of each immune checkpoint were defined as follows: PD-1 [11], PD-L1 [11], PD-L2 [11], CTLA-4 [12], TIM-3 [13], GAL-9 [14], CD272 [15], TIGIT [16], 2B4 [17], CD160 [18], KLRG-1 [19], and LAG-3 [20]. To monitor analytical performance, the HeLeNe principle (High-, Low- and No-expressor controls) was applied. Tonsils without pathology served as the reference tissue for lymphoid checkpoint antibodies, providing germinal-center B cells (high expressors) and mantle-zone B cells/epithelium (no expressors). A reagent-only slide, in which phosphate-buffered saline replaced the primary antibody, was included in every run to rule out non-specific background.

### 2.3. Statistical Analysis

Association between immune checkpoint molecules expression level and clinical and pathological variables was statistically examined correlation analysis and differences were assessed resorting to the *t*-test, after verifying the normality assumption. The association between histopathological variables was assessed using Cramer’s V statistic for all possible combinations. All analyses were conducted using IBM^®^ SPSS^®^ v28 software. Statistical significance was determined at a threshold of *p* < 0.05, and all tests were two-tailed.

## 3. Results and Discussion

### 3.1. Sample Demographic and Clinical Parameters

In this study involving 30 patients with CHB, we conducted a comprehensive assessment of demographic and clinical parameters (Table 1 and Table S1). Gender distribution revealed 20 male (66.7%) and 10 female (33.3%) participants. The average age of the sample was 51 years, with a standard deviation of 16.4 years. HBeAg testing indicated positivity in seven participants (25.9%) and negativity in 20 (74.1%). Notably, 10 patients exhibited negative HBV DNA (34.5%), while 9 were below 100,000 IU/mL (31.0%) and 10 above (34.5%). Additionally, 13 patients (43.3%) were on medication and 10 patients (33.3%) had progressed to hepatocellular carcinoma (HCC). In our sample of CHB patients, the average alanine aminotransferase (ALT) level was 102.5 U/L, with a standard deviation of 195.3 U/L. These findings offer valuable insights into the demographic and clinical characteristics of CHB patients.

### 3.2. Immune Checkpoint Immunohistochemical Evaluation

Following immunohistochemical staining of the various immune checkpoints, their distribution within the hepatic tissue was analyzed to elucidate the underlying interaction mechanisms. To account for inter-patient variability and enhance interpretability, a detailed summary of immune checkpoint expression per patient is provided in Supplementary Table S2, with a corresponding heatmap visualization included as Supplementary Figure S1.

**Figure 1:**
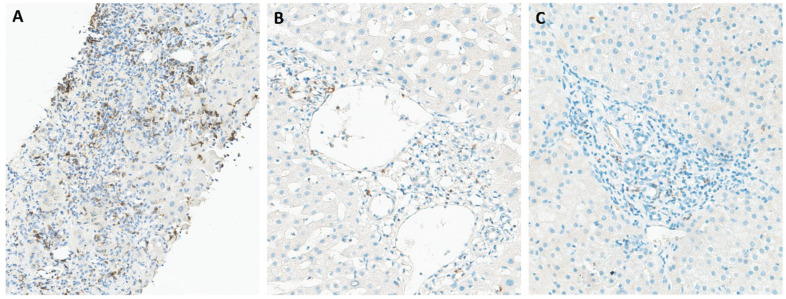
Immunohistochemical staining of liver tissues from patients with chronic hepatitis B for PD-1 (**A**), PD-L1 (**B**), and CTLA-4 (**C**). PD-1 is the most evident stain in portal tracts, with mild and focal staining for PD-L1 and CTLA-4.

Programmed cell death protein 1 (PD-1), also referred to as CD279, is an immune checkpoint receptor predominantly expressed on T cells, B cells, and NK cells. Its two ligands, PD-L1 (also known as B7-H1 or CD274) and PD-L2 (B7-DC or CD273), are expressed by various immune cells and tissues [21]. PD-L1 is notably upregulated by antigen-presenting cells (APCs) and non-lymphoid tissues, including the liver, while PD-L2 is primarily expressed by macrophages and dendritic cells (DCs) [22]. The main role of PD-1 is to regulate inflammatory responses during infections and prevent autoimmunity, by modulating the activity of effector T cells within peripheral tissues [23]. PD-1 was detected in 43% (13/30) of samples and its expression, predominantly observed in portal tracts, with additional presence in sinusoids (Figure 1A). Conversely, PD-L1 exhibited more focal staining patterns (Figure 1B), with only 10% (3/30) of patients being positive for this ligand. PD-L2 expression was absent from all liver samples obtained from CHB patients. Consistent with our findings, previous studies have reported predominant PD-1 expression in infiltrating lymphocytes within the portal area of the liver in patients with CHB [24,25,26]. Additionally, PD-1 expression was detected in the lobular region [24] and within the sinusoids [25]. In agreement with our findings, Wang et al. similarly reported weakly detectable PD-L1 and PD-L2 expression in liver tissues affected by hepatitis B [27]. They noted that cells expressing PD-L1 and PD-L2 were distinctly different from lymphocytes and hepatocytes, likely representing Kupffer cells (KCs) and liver sinusoidal endothelial cells (LSECs) [27]. Chen et al. associated the presence of PD-L1-positive cells with their localization near the portal area in the liver as well as the sinusoidal area [26]. While some studies have shown increased PD-L1 expression on KCs) in patients with CHB [24,28], others have reported no significant alterations in PD-L1 levels on KCs during HBV infection [26,27,29]. PD-L2-positive cells were infrequently observed in the liver tissues of patients with chronic hepatitis B [26]. Additionally, cells expressing PD-1, PD-L1, and PD-L2 cells were not present in the hepatic tissue from healthy controls [25,26].

CTLA-4 (cytotoxic T-lymphocyte-associated antigen 4), a significant immune checkpoint receptor, is often found on the surface of T cells and plays a key role in regulating T cell function [23]. Following antigen recognition, the expression of CTLA-4 is induced, resulting in the inhibition of T cell activation. CTLA-4 competes with CD28 for their ligands and transmits inhibitory signals to the T cell, thereby regulating T cell activation and maintaining immune homeostasis [30,31]. This regulatory function of CTLA-4 helps to prevent excessive immune responses by dampening signaling pathways involved in T cell activation. The immunohistochemical assessment revealed focal staining patterns for CTLA-4, with positive expression observed in only 2 out of the 30 liver samples analyzed. While Zhong et al. remains the sole study reporting CTLA-4 immunohistochemical evaluation in liver biopsies of CHB patients, their findings unveil a disease state-dependent expression of CTLA-4. Specifically, they highlight CTLA-4 overexpression during cirrhosis, contrasting with decreased expression in cases of active chronic hepatitis and asymptomatic carriers [32]. Interestingly, none of the positive patients in our sample were classified as having cirrhosis.

**Figure 2:**
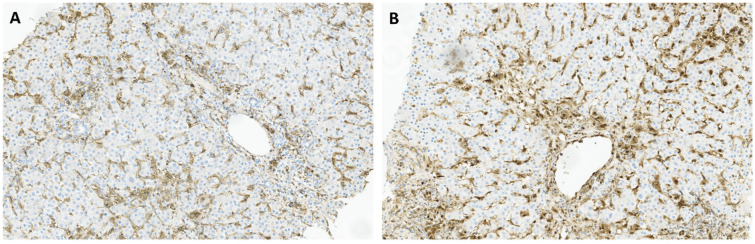
Immunohistochemical staining of liver tissues from patients with chronic hepatitis B for TIM-3 (**A**), and GAL-9 (**B**), with a predominant staining in inflammatory cells and sinusoidal tracts.

TIM-3 and galectin-9, (GAL-9) its ligand, have gained increasing attention as potential targets for some infectious disease and cancer immunotherapy. TIM-3 is constitutively expressed on several immune cell types, such as monocytes, macrophages and DCs, as well as CD4+ and CD8+ T cells [33]. GAL-9 can be found in many organs, including lymphoid tissues, the small intestine, and the liver. The TIM-3:GAL-9 pathway plays a key role in controlling T cell proliferation and tolerance, since their interaction leads to T cell inhibition and apoptosis [34]. TIM-3 emerged as the principal immune checkpoint in our study, with only one negative sample out of 30 patients, while GAL-9 was found in 43% (13/30) of the samples. Additionally, TIM-3 immunohistochemical staining revealed predominant expression in portal tracts and sinusoids (Figure 2A), whereas GAL-9 exhibited a more diffuse expression pattern, including inflammatory cells within sinusoids (Figure 2B). Previous studies have reported TIM-3 overexpression on CD4+ and CD8+ T cells, in particular within the intrahepatic tissue of CHB patients [35]. Additionally, up-regulation of TIM-3 expression was observed on peripheral blood mononuclear cells (PBMCs) and liver infiltrating lymphocytes (LILs) in patients with CHB, compared to controls [36]. Moreover, researchers reported a pan-lobular expression of GAL-9, as well as increased levels in the liver of patients with active disease, particularly within Kupffer cells in hepatic sinusoids [35]. This distribution of GAL-9 positions it ideally to induce functional inactivation and deletion of HBV-specific CD8 T cells expressing TIM-3 as they migrate into hepatic tissue [37]. The preferential expression of GAL-9 in the liver and its proximity to sinusoids suggest that interactions between TIM-3 and GAL-9 may play a crucial role in HBV infection. Considering the inhibitory role of TIM-3 in antiviral immune responses, it emerges as a promising target for HBV infection treatment [33]. Continued investigation into the TIM-3:GAL-9 pathway and its immunoregulatory functions holds the potential to unveil innovative therapeutic approaches against chronic HBV infection.

**Figure 3:**
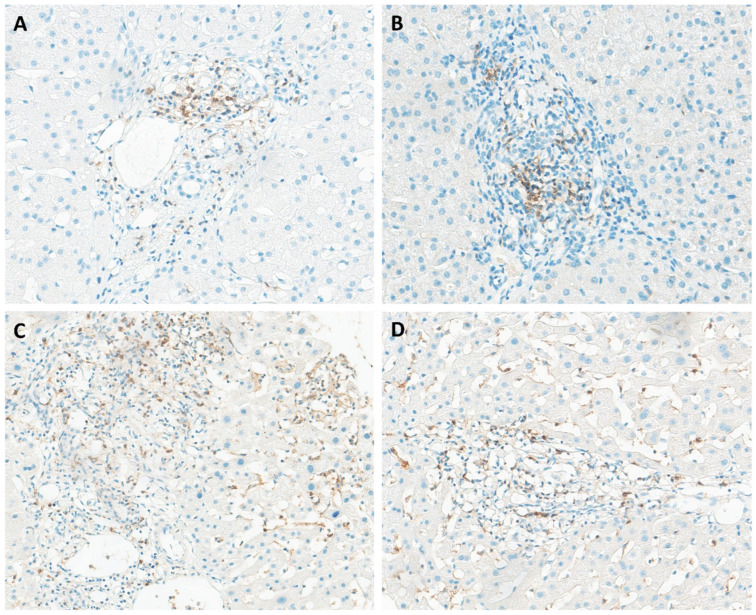
Immunohistochemical staining of liver tissues from patients with chronic hepatitis B for CD272 (**A**), TIGIT (**B**), and 2B4 (**C**,**D**). All markers exhibited expression in portal tracts, with 2B4 also showing expression in sinusoidal inflammatory cells.

The receptor CD272, also known as B and T lymphocyte attenuator (BTLA), plays a pivotal role in a complex receptor-ligand network involving the herpesvirus entry mediator (HVEM). CD272 is prominently expressed on activated T cells and resting B cells, with lower expression observed on NK cells and some APCs, suggesting its involvement in transmitting inhibitory signals to various immune cell populations [38]. CD272 immunohistochemical evaluation revealed its presence in over half of the liver samples (17/30). Moreover, our analysis revealed moderate and heterogeneous staining of CD272 in inflammatory cells, particularly within lymphoid aggregates, with focal expression observed in portal inflammatory cells in some patients (Figure 3A). Interestingly, previous findings indicated the absence of CD272 in normal liver tissues and in the samples from CHB patients, while strong expression of CD272 was observed in benign bile ducts and infiltrating inflammatory cells in cases of HBV-ACLF [39].

The T cell immunoglobulin and immunoreceptor tyrosine-based inhibitory motif domain, known as TIGIT, represents a co-inhibitory receptor expressed by NK cells and various T cell subsets, including memory T cells and regulatory T cells (Tregs) [40]. Upon interaction with its ligand CD155, expressed on the surface of APCs, TIGIT initiates negative signaling pathways that suppress T cell activity, mediated through the upregulation of IL-10 secretion [41]. Immunohistochemical evaluation of TIGIT in liver tissue from patients with chronic hepatitis B has not been previously documented in the literature. TIGIT expression was detected in 13% of the samples (4/30), primarily localized to portal tracts (Figure 3B). Although the role of TIGIT in HBV-related immune tolerance remains relatively unexplored, recent studies have started to illuminate this area. For example, Zong et al. observed a significantly higher percentage of TIGIT+CD8+ T cells in the blood of CHB patients compared to healthy adults using flow cytometry [40].

CD244, also known as 2B4, can be found in innate immune cells such as NK cells, as well as CD8+ T cells. The interaction between 2B4 and its ligand CD48 can impair the development of T cell function, resulting in inhibitory signals. This study constitutes the first immunohistochemical report of 2B4 receptor expression in the liver of CHB patients. 2B4 expression was observed in 47% (14/30) of CHB patients, primarily localized within portal spaces and sinusoidal inflammatory cells (Figure 3C,D). Interestingly, the expression of 2B4 tends to be elevated in intrahepatic CD8+ T cells compared to peripheral ones, indicating a more severe state of immune exhaustion in liver-infiltrating CD8+ T cells [42]. Furthermore, researchers have proposed that the upregulation of 2B4 is linked to PD-1 overexpression, suggesting that modulating 2B4 on its own may have fewer profound effects on T cell restoration compared to targeting other co-inhibitory receptors [22].

Although our study marks the first immunohistochemical evaluation of CD160, LAG-3, and KLRG-1 expression in liver biopsies of CHB patients, no subjects showed expression of these immune checkpoints.

### 3.3. Association Between Demographic and Clinical Data and Histopathological Variables

The demographic and clinical data of the sample were described using absolute and relative frequencies for qualitative variables, and mean, standard deviation, median, and percentiles 25 and 75 for quantitative variables (Table S1). In order to assess if the frequency distribution was homogeneous in the sample, the binomial test was used for binary variables, and the chi-squared goodness of fit test assuming equal frequencies was used for categorical variables with more than two categories. This assessment allowed us to identify variables that exhibited heterogeneity in the sample and therefore study them in more detail. The measured immunohistochemical parameters were described using absolute and relative frequencies (Table S3). Similarly, the binomial and chi-squared goodness of fit tests were employed to detect parameters that exhibited heterogeneity within the group. The relationship between ALT values and histological parameters was analyzed using linear regression.

The distribution of age among CHB patients is symmetrical, with a mean of 51 years and a standard deviation of 16.4 years. It can be observed that there is homogeneity in most clinical parameters among patients with CHB, except for the HBeAg, which is more frequently negative. As expected, there is a strongly positively skewed distribution of ALT values, with a median of 40.0, a 25th percentile of 25.0, and a 75th percentile value of 75.8 (the 90th percentile is 301.3, and the maximum is 988.0). The parameters PD-L2, CD160, LAG-3, and KLRG-1 do not vary across the entire sample, as they are negative for all subjects. Regarding the CD272 parameter, although it is not the same among subjects, the difference in proportions is not statistically significant (*p* = 0.141). In relation to the remaining parameters, statistical significance is observed regarding the difference in frequencies.

A linear model was adjusted using the stepwise method between ALT values and different immunohistochemical parameters. The obtained model is statistically significant (*p* < 0.001), explaining approximately 58% of the variance (Adjusted R-squared = 0.577). Only some categories have been found to be statistically significant (Table S4). The results can be interpreted as follows: when PD-1 is <10%, ALT increases by an average of 118.2 units compared to PD-1 being negative; when TIM-3 is ≥10%, ALT increases by an average of 449.9 units compared to TIM-3 being negative; and when GAL-9 is scored as 2, ALT decreases by an average of 151.1 units compared to GAL-9 being negative.

This result suggests that there may be a correlation between lower levels of PD-1 expression and elevated levels of ALT, an enzyme often associated with liver damage or inflammation. When PD-1 staining was <10%, ALT levels rose by an average of 118.2 IU/L versus PD-1-negative cases, indicating that low-level PD-1 induction may coincide with heightened immune-mediated liver damage. In contrast, the small ≥ 10% subgroup did not differ significantly from the PD-1-negative group. We speculate that borderline PD-1 up-regulation reflects recently activated T cells capable of cytolytic activity, whereas higher PD-1 density marks deeper functional exhaustion that limits further ALT elevation.

Interestingly, a study by Wang et al. observed that the expression of PD-1 in liver tissues from patients with hepatitis B did not show any correlation with age, gender, ALT levels, or histological activity index (HAI). Similarly, the expression of PD-L1 and PD-L2 in liver tissues from hepatitis B patients did not exhibit any association with age, gender, or levels of ALT and total bilirubin (TB) [27]. In contrast, Xie et al. noticed an up-regulation of PD-1 and PD-L1 expression in livers affected by CHB, and their expression levels demonstrate a significant correlation with viral load, inflammatory reactions, and the progression of the disease [43].

Regarding TIM-3, when its expression is 10% or higher, there is, on average, a significant increase of 449.9 units in ALT levels compared to when TIM-3 expression is negative. This indicates that higher levels of TIM-3 expression may be associated with more severe liver injury or inflammation, as indicated by the elevation in ALT levels. However, when GAL-9 is scored as 2, there is an average decrease of 151.1 units in ALT levels compared to when GAL-9 expression is negative. This could indicate that moderate GAL-9 expression levels may be associated with decreased liver injury or inflammation, as indicated by the reduction in ALT levels. Given the impact of the TIM-3:GAL-9 pathway on antiviral immune responses, our findings underscore the intricate role of this pathway in hepatic immune exhaustion in CHB. This emphasizes the importance of further studies into the immunoregulatory functions of the TIM-3:GAL-9 pathway. Such investigations hold promise for the development of novel therapeutic strategies aimed at combating chronic HBV infection.

From a therapeutic standpoint, disrupting the TIM-3:GAL-9 axis offers several clinically relevant benefits for CHB. First, it can reinvigorate exhausted HBV-specific CD8+ T cells. In vitro blockade of TIM-3 restores proliferation, IFN-γ/TNF-α production and cytotoxicity, even when PD-1 signaling remains active [35]. Enhancing this arm of antiviral immunity may facilitate clearance of cccDNA-harboring hepatocytes and advance the goal of a functional cure (HBsAg loss). Second, TIM-3:GAL-9 interference may reshape the intrahepatic immune milieu and slow fibrosis progression. GAL-9 drives local immunological tolerance; interrupting its interaction with TIM-3 is predicted to tip the balance toward controlled antiviral inflammation, thereby reducing long-term fibrogenesis and hepatocarcinogenesis. Third, TIM-3 blockade is a rational partner for existing therapies. Dual inhibition of TIM-3 and PD-1 may produce synergistic antiviral effects, while sequential regimens (nucleos(t)ide analog suppression followed by TIM-3 inhibition) may maximize efficacy and minimize immune-mediated flares. Safety remains paramount—ALT flares can occur when immune brakes are released—but careful patient selection (compensated liver disease, close biochemical monitoring) and staggered dosing schedules should mitigate risk [44]. Several TIM-3–directed biologics have now advanced into first-in-human studies, demonstrating that clinical manipulation of this pathway is technically feasible. A prominent example is LY3415244, a bispecific IgG4 that simultaneously targets TIM-3 and PD-L1 in patients with heavily pre-treated solid tumors [45]. Additionally, LB1410, a tetravalent bispecific antibody that co-engages TIM-3 and PD-1, is currently enrolling patients with advanced solid malignancies or lymphoma in a Phase I, first-in-human trial (NCT05820238) [46]. Although these strategies are oncology-focused, they provide a clinically validated blueprint for TIM-3 intervention that could be repurposed for CHB. Together, these considerations highlight TIM-3:GAL-9 as a promising yet carefully calibrated target in future HBV immunotherapy trials [47].

The clinical parameters Stage, HAI, HCC, and HBV DNA were assessed using logistic regression and multinomial models depending on the nature of the variable, with CD272, 2B4, GAL-9, PD-1, and TIM-3 as independent variables. The regression models tested for the clinical variables Stage, HAI, HCC, and HBV DNA were not statistically significant.

### 3.4. Association Between Histopathological Variables

To analyze potential associations between the histopathological variables, which are categorical variables, the Cramer’s V statistic was calculated for all combinations. In order to reduce the number of statistical tests and thus mitigate the issue of multiple comparisons, only pairs of variables with a Cramer’s V value greater than 0.7 underwent the chi-squared test (Table S5). From the table, it can be observed that only the pairs CD272/TIGIT and GAL-9/CTLA-4 have Cramer’s V values greater than 0.7. There is a statistically significant association (Chi-square test, *p* < 0.001) between CD272 and TIGIT (Table S6), as well as between GAL-9 and CTLA-4 (Table S7).

This finding indicates that there is a strong relationship between the expression of CD272 and TIGIT, as well as between GAL-9 and CTLA-4, within the studied population. In other words, the presence or absence of one marker (CD272 or GAL-9) is closely related to the presence or absence of the other marker (TIGIT or CTLA-4), respectively. These associations could imply potential interactions or shared regulatory mechanisms between the pairs of immune checkpoints in a CHB scenario. Further investigation into these relationships may provide insights into the underlying immune pathways and their involvement in the pathogenesis of the CHB.

## 4. Conclusions

While recent studies offer promise for CHB therapies, there remains a crucial need for further research to refine treatment strategies, pinpoint biomarkers for patient selection, and understand potential immune-related adverse events. Among these, immune checkpoint inhibitors stand out as a particularly promising avenue for CHB treatment, with the potential to revitalize functional antiviral immune responses and achieve lasting viral control. Our study highlights the predominant localization of immune checkpoint expression in portal tracts and sinusoids in patients with CHB. Importantly, we observed a correlation between the expression levels of PD-1, TIM-3, and GAL-9 with ALT levels, suggesting a potential association between immune checkpoint expression and liver injury in CHB. Furthermore, strong relationships were identified between the expression of CD272 and TIGIT, as well as between GAL-9 and CTLA-4 within the studied population. Notably, the TIM-3:GAL-9 pathway emerges as a key player in CHB immunopathogenesis, underscoring the importance of exploring combination therapies that target this pathway alongside conventional treatments. By delving deeper into these issues, we can unlock new insights into CHB management and pave the way for more effective and personalized therapeutic approaches in the future.

## Figures and Tables

**Table 1:** Chronic hepatitis B patient information and sample characteristics. ALT measured international units per liter (IU/L) and HBV-DNA expressed in international units per milliliter (IU/mL). TDF = Tenofovir Disoproxil Fumarate; ETV = Entecavir; N.A. = Not Available.

No.	Gender	Age	HBeAg	HBV DNA	Medication	HCC	ALT	Stage	HAI
N1	M	29	Pos.	>1,000,000	No	No	988	F2	5
N2	F	29	Neg.	<10	No	No	28	F1	1
N3	M	51	N.A.	>2,000,000	No	No	N.A.	F4	5
N4	M	78	Pos.	>25,000,000	No	No	77	F4	N.A.
N5	F	55	Neg.	3943	No	No	25	F1	3
N6	F	47	Neg.	198	ETV	No	28	F2	1
N7	M	51	Neg.	78	No	Yes	72	F4	5
N8	F	66	Pos.	2595	No	No	53	F4	1
N9	F	59	Neg.	742,173	TDF	Yes	85	F2	1
N10	M	12	Pos.	>1,700,000	No	No	92	N.A.	N.A.
N11	M	N.A.	N.A.	N.A.	N.A.	N.A.	N.A.	N.A.	N.A.
N12	M	51	Neg.	100,273	No	No	430	F1	6
N13	M	54	Neg.	Neg.	TDF	No	35	F2	2
N14	M	44	Neg.	4204	No	No	25	F0	0
N15	M	38	Pos.	<20	TDF	No	60	F2	1
N16	M	63	Neg.	1,250,750	No	Yes	49	F4	2
N17	M	70	Neg.	Neg.	ETV	Yes	27	F1	1
N18	M	43	Neg.	121	No	No	64	F0	0
N19	F	25	Neg.	Neg.	No	No	13	F0	0
N20	M	59	Neg.	Neg.	No	No	287	N.A.	N.A.
N21	M	34	Neg.	Neg.	TDF	No	44	F3	0
N22	M	78	Neg.	4218	No	Yes	16	F4	N.A.
N23	M	65	Pos.	>170,000,000	TDF/ETV	Yes	59	F0	1
N24	F	46	Neg.	Neg.	ETV	No	16	F1	1
N25	M	56	Neg.	Neg.	ETV	Yes	25	F4	N.A.
N26	F	40	Pos.	165,000	TDF/ETV	No	160	F4	N.A.
N27	F	36	N.A.	Neg.	No	Yes	36	F4	0
N28	M	59	Neg.	Neg.	TDF	No	19	F3	0
N29	M	65	Neg.	121,450	TDF	Yes	25	F4	N.A.
N30	F	76	Neg.	Neg.	TDF	Yes	32	F1	1

## Data Availability

The original contributions presented in this study are included in the article. Further inquiries can be directed to the corresponding author.

## Supplementary Materials

The following supporting information can be downloaded at: https://www.mdpi.com/article/10.3390/pathogens14060596/s1, Table S1: The demographic and clinical data of the sample. Quantitative variables: mean (standard deviation) median percentile 25/percentile 75; Table S2: Immunohistochemical data for individual patients with CHB; Figure S1: Heatmap summarizing immune checkpoint expression profiles across individual patients; Table S3: The immunohistochemical parameters of the sample; Table S4: Regression coefficients results for the different independent variables; Table S5: Cramer’s V values for pairs of histopathological variables; Table S6: Cross-distribution of values between CD272 and TIGIT; Table S7: Cross-distribution of values between GAL-9 and CTLA-4.
