# Supplementary material for: Immunohistochemical Profiling of Immune Checkpoints in Chronic Hepatitis B Liver Tissue
Source: Pathogens. 2025 Jun 18;14(6):596. doi: 10.3390/pathogens14060596 (PMC12196340; doi:10.3390/pathogens14060596)
Supplement: Supplementary file 1 [file pathogens-14-00596-s001.zip › pathogens-3684506-supplementary.pdf]

Supplementary Materials

Table S1: The demographic and clinical data of the sample. Quantitative variables: mean (standard deviation) median percentile 25/percentile 75. § Binomial test. # Chi-squared goodness of fit test.

Variable		Frequency	p
Gender	M	20 (66.7 %)	0.100§
	F	10 (33.3 %)	
Age	51.0 (16.4)<br>51.0 39.0/64.0		
HBeAg	Neg.	20 (74.1 %)	0.021§
	Pos.	7 (25.9 %)	
HBV DNA	Neg.	10 (34.5 %)	0.966#
	< 100000	9 (31.0 %)	
	≥ 100000	10 (34.5 %)	
Medication	No	17 (56.7 %)	0.584§
	Yes	13 (43.3 %)	
HCC	No	20 (66.7 %)	0.100§
	Yes	10 (33.3 %)	
ALT	102.5 (195.3)<br>40.0 25.0/75.8		
Stage	F0	4 (14.8 %)	0.164#
	F1	6 (22.2 %)	
	F2	5 (18.5 %)	
	F3	2 (7.4 %)	
	F4	10 (37.0 %)	
HAI	0	6 (27.3 %)	0.094#
	1-4	12 (54.5 %)	
	> 4	4 (18.2 %)	

Table S2: Immunohistochemical data for individual patients with CHB. The classification of immune checkpoint expression levels was defined according to the criteria described in Section 2.2.

No.	PD-L2	CD272	CD160	2B4	GAL-9	PD-1	CTLA-4	TIM-3	LAG-3	KLRG-1	PD-L1	TIGIT
**N1**	Neg.	Neg.	Neg.	Neg.	Neg.	<5 %	Neg.	5x3	Neg.	Neg.	Neg.	Neg.
**N2**	Neg.	Neg.	Neg.	Focal expression in inflammatory cells in sinusoids and portal spaces	Neg.	<5 %	Neg.	1x1	Neg.	Neg.	Neg.	Neg.
**N3**	Neg.	Weak and focal	Neg.	Neg.	Neg.	Neg.	Neg.	2x2	Neg.	Neg.	Neg.	Neg.
**N4**	Neg.	Weak and focal	Neg.	Focal expression in portal spaces	Focal<br>Score 1	Neg.	Neg.	2x2	Neg.	Neg.	Neg.	Neg.
**N5**	Neg.	Weak and focal	Neg.	Neg.	Moderate expression in inflammatory cells in sinusoids and follicles<br>Score 2	10-20 %	Neg.	4x3	Neg.	Neg.	Neg.	Neg.
**N6**	Neg.	Weak and focal	Neg.	Neg.	Neg.	Neg.	Neg.	Neg.	Neg.	Neg.	Neg.	Neg.
**N7**	Neg.	Moderate and heterogeneous in inflammatory cells, mainly in lymphoid aggregates	Neg.	Focal expression in inflammatory cells in the sinusoids	Focal expression in some germinal centers and in sinusoidal inflammatory cells<br>Score 1	5 %	Neg.	1x1	Neg.	Neg.	Neg.	Score 1
**N8**	Neg.	Weak and focal	Neg.	Focal expression in inflammatory cells in the sinusoids	Neg.	<5 %	Neg.	2x1	Neg.	Neg.	Neg.	Neg.
**N9**	Neg.	Moderate and heterogeneous in inflammatory cells, mainly in lymphoid aggregates	Neg.	Moderate expression in inflammatory cells in the sinusoids	Score 2 in inflammatory cells in sinusoids and follicles, focally with score 3	5-10 %	<1%<br>Score 1	4x2	Neg.	Neg.	Neg.	Score 1
**N10**	Neg.	Moderate and heterogeneous in inflammatory cells, mainly in lymphoid aggregates	Neg.	Neg.	Focal<br>Score 1	Neg.	Neg.	3x3	Neg.	Neg.	10 %	Score 1
**N11**	Neg.	Focal expression in portal inflammatory cells	Neg.	Neg.	Score 2 in inflammatory cells in sinusoids and follicles, focally with score 3	Neg.	Neg.	5x2	Neg.	Neg.	20 %	Neg.
**N12**	Neg.	Focal to moderate expression in portal inflammatory cells	Neg.	Focal expression in inflammatory cells in the sinusoids	Score 3	Neg.	<5%<br>Score 1	5x3	Neg.	Neg.	20 %	Score 1
**N13**	Neg.	Focal expression in portal inflammatory cells	Neg.	Neg.	Neg.	Neg.	Neg.	1x1	Neg.	Neg.	Neg.	Neg.
**N14**	Neg.	Neg.	Neg.	Focal expression in portal spaces	Neg.	Neg.	Neg.	1x1	Neg.	Neg.	Neg.	Neg.
**N15**	Neg.	Neg.	Neg.	Neg.	Neg.	Neg.	Neg.	1x1	Neg.	Neg.	Neg.	Neg.
**N16**	Neg.	Neg.	Neg.	Focal expression in portal spaces	Neg.	10-20 %	Neg.	1x1	Neg.	Neg.	Neg.	Neg.

**N17**	Neg.	Neg.	Neg.	Neg.	Score 2	Neg.	Neg.	1x1	Neg.	Neg.	Neg.	Neg.
**N18**	Neg.	Focal expression in portal inflammatory cells	Neg.	Moderate expression in inflammatory cells in sinusoids and portal spaces	Neg.	Neg.	Neg.	3x1	Neg.	Neg.	Neg.	Neg.
**N19**	Neg.	Neg.	Neg.	Focal expression in inflammatory cells in the sinusoids	Neg.	Neg.	Neg.	3x1	Neg.	Neg.	Neg.	Neg.
**N20**	Neg.	Focal to moderate expression in portal inflammatory cells	Neg.	Neg.	Neg.	<1 %	Neg.	2x1	Neg.	Neg.	Neg.	Neg.
**N21**	Neg.	Focal expression in portal inflammatory cells	Neg.	Focal expression in inflammatory cells in the sinusoids	Score 2	10-20 %	Neg.	3x1	Neg.	Neg.	Neg.	Neg.
**N22**	Neg.	Focal expression in portal inflammatory cells	Neg.	Neg.	Neg.	Neg.	Neg.	1x1	Neg.	Neg.	Neg.	Neg.
**N23**	Neg.	Neg.	Neg.	Neg.	Score 1	Neg.	Neg.	2x1	Neg.	Neg.	Neg.	Neg.
**N24**	Neg.	Focal expression in portal inflammatory cells	Neg.	Neg.	Score 1	Neg.	Neg.	1x1	Neg.	Neg.	Neg.	Neg.
**N25**	Neg.	Focal expression in portal inflammatory cells	Neg.	Focal expression in inflammatory cells in sinusoids and portal spaces	Neg.	20-30 %	Neg.	4x1	Neg.	Neg.	Neg.	Neg.
**N26**	Neg.	Neg.	Neg.	Neg.	Neg.	10 %	Neg.	3x1	Neg.	Neg.	Neg.	Neg.
**N27**	Neg.	Neg.	Neg.	Neg.	Score 2	<1 %	Neg.	4x2	Neg.	Neg.	Neg.	Neg.
**N28**	Neg.	Neg.	Neg.	Neg.	Neg.	Neg.	Neg.	3x1	Neg.	Neg.	Neg.	Neg.
**N29**	Neg.	Neg.	Neg.	Moderate expression in inflammatory cells in the sinusoids	Neg.	Neg.	Neg.	1x1	Neg.	Neg.	Neg.	Neg.
**N30**	Neg.	Focal to moderate expression in portal inflammatory cells	Neg.	Focal expression in inflammatory cells in the sinusoids	Score 1	<5 %	Neg.	3x1	Neg.	Neg.	Neg.	Neg.

Figure S1: Heatmap summarizing immune checkpoint expression profiles across individual patients. Color coding represents expression intensity: red = negative; orange = low; yellow = intermediate; green = high. Rows correspond to individual patients, and columns to specific immune checkpoint molecules.

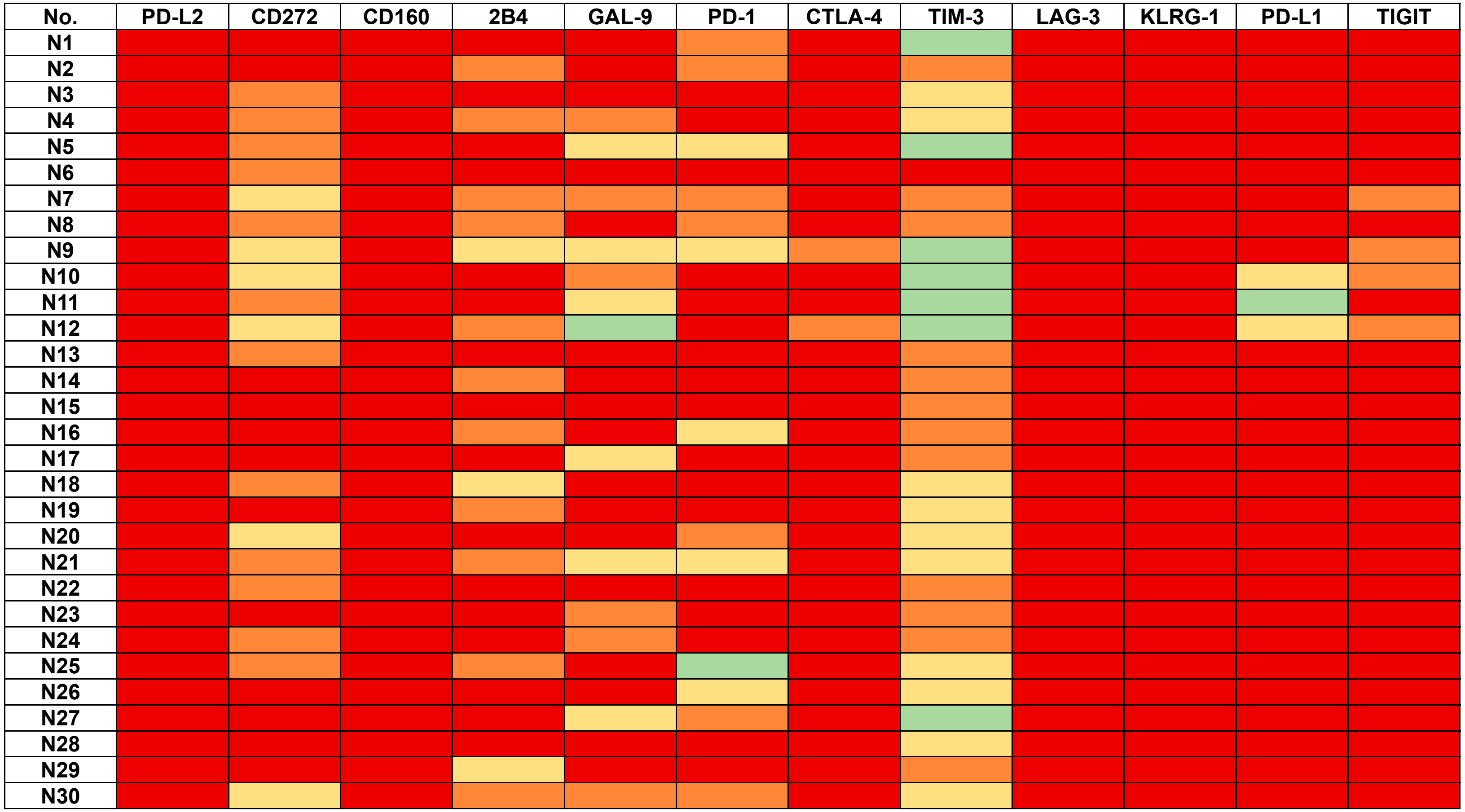

Table S3: The immunohistochemical parameters of the sample. § Binomial test. # Chi-squared goodness of fit test.

Parameter		Frequency	p
PD-L2	Neg.	30 (100.0 %)	
	Pos.	0 (0.0 %)	
CD272	Neg.	13 (43.3 %)	0.141
	Weak	5 (16.7 %)	
	Moderate	6 (20.0 %)	
	Intermediate	6 (20.0 %)	
CD160	Neg.	30 (100.0 %)	
	Pos.	0 (0.0 %)	
2B4	Neg.	16 (53.3 %)	**0.014**
	Weak	0 (0.0 %)	
	Moderate	11 (36.7 %)	
	Intermediate	3 (10.0 %)	
GAL-9	Neg.	17 (56.7 %)	**<0.001**
	Score 1	6 (20.0 %)	
	Score 2	4 (13.3 %)	
	Score 3	3 (10.0 %)	
PD-1	Neg.	17 (56.7 %)	**<0.001**
	< 10 %	8 (26.7 %)	
	[10, 20[%	4 (13.3 %)	
	≥ 20 %	1 (3.3 %)	
CTLA-4	Neg.	28 (93.3 %)	**<0.001**
	< 10 %	2 (6.7 %)	
TIM-3	Neg.	1 (3.3 %)	**<0.001**
	< 10 %	22 (73.3 %)	
	[5, 10[%	3 (10.0 %)	
	≥ 10 %	4 (13.3 %)	
LAG-3	Neg.	30 (100.0 %)	
	Pos.	0 (0.0 %)	
KLRG-1	Neg.	30 (100.0 %)	
	Pos.	0 (0.0 %)	
PD-L1	Neg.	27 (90.0 %)	**<0.001**
	< 10 %	0 (0.0 %)	
	[10, 20[%	1 (3.3 %)	
	≥ 20 %	2 (6.7 %)	
TIGIT	Neg.	26 (86.7 %)	**<0.001**
	Score 1	4 (13.3 %)	

Table S4: Regression coefficients results for the different independent variables.

Variable	Coefficient	p
Constant	42.1	0.060
TIM-3 [≥ 10%]	449.9	0.000
PD-1 [< 10%]	118.2	0.478
GAL-9 [Score2]	-151.1	<0.001

Table S5: Cramer's V values for pairs of histopathological variables.

	CD272	2B4	GAL-9	PD-1	CTLA-4	TIM-3	PD-L1	TIGIT
CD272		0.312	0.402	0.323	0.535	0.348	0.356	0.784
2B4			0.249	0.271	0.395	0.299	0.143	0.255
GAL-9				0.281	0.802	0.43	0.624	0.65
PD-1					0.164	0.215	0.206	0.241
CTLA-4						0.491	0.465	0.681
TIM-3							0.621	0.582
PD-L1								0.567

Table S6: Cross-distribution of values between CD272 and TIGIT.

		TIGIT	
		Neg.	Score 1
CD272	Neg.	13	0
	Weak	5	0
	Moderate	6	0
	Intermediate	2	4

Table S7: Cross-distribution of values between GAL-9 and CTLA-4.

		CTLA-4	
		Neg.	< 10 %
GAL-9	Neg.	17	0
	Score 1	6	0
	Score 2	4	0
	Score 3	1	2
